# Remarks on Intestinal Parasites

**Published:** 1882-06-20

**Authors:** F. P. Henry


### REMARKS ON INTESTINAL PARASITES.

BY F. P. HENRY, M. D.

Read before the Clinical Section of the Philadelphia County Medical Society, January 31, 1882.

The subject of human parasites, upon which I was requested, at rather short notice, to make some remarks this evening, is so comprehensive that, were I to undertake to refer to them all, within the limit of time allotted me, I could do little more than mention their names and those of the diseases to which they give rise. I have therefore decided to confine myself to a few general observations upon those varieties of worms which are most frequently found in the intestines of man. Of these the principal are the *Ascaris lumbricoides*, the *Oxyuris vermicularis*, and the different kinds of tape-worm. The first two are by far the most common, owing to their universal prevalence and the readiness with which specimens are obtained, we are thoroughly acquainted with their anatomy, both as embryos and as mature individuals. Nevertheless, the most competent helminthologists acknowledge their complete ignorance of the manner in which the lumbricoid worm is first introduced into the system. Leuckart has fed dogs, pigs rabbits and mice with the ripe eggs of the *Ascaris lumbricoides*, and has made similar experiments on man, in both cases with negative results. It is probable that these worms are introduced into the stomach in an embryonic form. Niemeyer has suggested the possibility of their finding entrance into the system through the use of bad flour, and refers to the observations of Stein, who found entozoa in weevils.

The *Oxyuris vermicularis* is developed directly from the egg, and this is one of the reasons of its persistent stay in the intestines, for by self infection the eggs are constantly swallowed by children and others who are careless in regard to habits of cleanliness. Self-infection is caused by scratching the anus and neighboring parts and subsequently conveying the eggs into the mouth. This may happen in the case of persons who are tolerably cleanly in their habits; for Zenker has frequently found mature eggs under the nails of those afflicted with these parasites.

It is thought by many that the principal habitat of th *Oxyuris* is the rectum, and this theory has led to a treatment that is at best but palliative. "The generally-prevalent idea," says Heller, "and that which is upheld in all the books, that the *Oxyuris* inhabits the rectum, is entirely false." The mature males inhabit chiefly the small intestine; the pregnant females chiefly the cæcum, where they remain until they are distended with eggs. They then descend to the rectum and deposit their eggs. The young, when hatched, immediately migrate to the small intestine.

The treatment of the *Oxyuris* recommended by Heller is one of saline catharsis, and of enemata so administered as to reach the cæcum. The cathartic part of this treatment is based upon the observation that in choleraic conditions vast quantities of parasitic ova are often expelled. The appearance of these ova in the stools of cholera patients led to a curious blunder both in Germany and England, where they were regarded as a fungus peculiar to that disease. Another species of worm, the *Trichocephalus dispar*, having been frequently found in the stools of typhoid fever patients, has been regarded by some as the cause of that affection. The symptoms due to the presence of the round worm scarcely suffice for a diagnosis. There can be no doubt that in some cases grave disturbance of the nervous system, in others intestinal catarrh, is casued by them, while in perhaps the most numerous class of cases they give rise to no symptoms that cannot be referred to ordinary causes.

Formerly it was the custom to attribute almost every digestive disorder occurring during childhood to the presence of worms. Now there appears to be a tendency to go to the opposite extreme, and to deny that they are the cause of any symptoms whatever. Heller remarks upon this tendency, and warns against it, although, says he, "there is little likelihood of our going so far as to look on them as the guardian angels of thildren, ever ready to help them in their time of need."

The treatment of lumbricoid worms is too well known for me to comment upon it. There is one point concerning it, however. which seems to me of some interest, and that is the fact that fractional grain doses of santonin retain their full activity after having passed through the stomach. Many physicians are sceptical as to the effect of small doses of drugs administered with a view to produce an alterative effect upon the small and large intestine; but facts like that above given should induce them to be cautious about rejecting any remedy upon mere hypothetical grounds.

The most interesting fact concerning the tape-worm is the complexity of its mode of existence. Its ova, being discharged from the intestine of man, are swallowed by another animal, in whose tissues they reach the larval stage of their existence. These larvæ, being in turn swallowed by man, reach the mature stage of their development in his intestine. This cyclical method of growth is in the highest degree opposed to the existence of the parasite, and is counterbalanced by its enormous fertility. According to E. Wagner, "in the case of the tape-worm, out of eighty-five millions of eggs, only one is developed again into a tape-worm."

Though many species of tape-worm are known to exist, four only are of interest and importance to the practical physician: these are, *Tœnia solium*, *Tœnia medio-conellata*, *Tœnia echinoccus*, and *Bothriocephialus lotus*.

Of these, the designation of the first is very ill chosen, indeed, I have found it impossible to ascertain what Linnæus intended by it.

If, as some think, he means solitary,—an absurd supposition, in my opinion,—he must have been mistaken as to the habits of this parasite ; for when a number of tape-worms inhabit the intestine they are almost invariably of the *solium* variety. I have consulted a distinguished classical scholar of this city, a member of our own profession, who acknowledges his inability to interpret the term *solium* as used in connection with *Tænia.* Such, however, is the weight of authority that, notwithstanding the obscurity surrounding this word, I shall continue to stultify myself by its employment.

Heller objects to the term *medio-conellata* applied to the second variety, on the ground that it is founded upon an erroneous anatomical idea; and employs instead the term *saginata*, stout or well fed, which it had originally received from Goeze.

The diagnosis as to the kind of tapeworm present in a case may be made by inspection of the segments voided per rectum. It is important that the variety of worm be clearly ascertained, for nervous symptoms occurring in an individual who is or has been the host of the *Tænia solium* should lead to the suspicion of the presence of the tape-worm larva, the *Cysticercus cellulosæ*, in some portion of the nerve-centres.

The chief seal of the *Cysticercus* is the intermuscular connective tissue, after which come the brain and the eye. In the latter situation Von Graefe was able in four cases to watch the development of the entoozoon from the time of its appearance beneath the retina, which it pushes before it, causing a more or less extensive detachment, until its eruption into the vitreous humor, which, in the majority of cases, it makes its seat. An interesting case of intraocular cysticerus recently occurred in the practice of Dr. James E. Garretson, of this city, and is reported by Dr. C. S. Turnbull in vol. xii. of the Transactions of the State Medical Society, to which I rêfe those interested in the subject. Another case of suspended intraocular cysticercus was recently exhibited by Dr. Garretson to the Pathological Society of Philadelphia. I had an opportunity of seeing it, and was struck with the remarkable similarity, both in size and shape, which it bore to a cysticercus. In reply to a note asking for the subsequent history of this case, Dr. Garretson informs me that he has lost sight of the patient.

When the *Tænia solium* and its cysticercus are present in the same case, what is the source of the latter ? This is an interesting question and one which has received different answers. Self-infection would be the answer of Heller, while Roberts considers that they are derived from independent sources. The former method of the introduction of the cyst into the solid tissues of one who is the host of a tape-worm seems to me the most probable. Self-infection may occur by detached segments being forced upward, during the act of vomiting, into the stomach, where, undergoing digestion by the gastric juice, the eggs are liberated and are developed into cysticerci. Or it is perhaps permissible to suppose that such action may take place in the upper portion of the small intestine, within the sphere of the pancreatic secretion, which fluid, as is well known, contains a ferment called trypsin, which is capable of digesting proteids in an alkaline medium. The practical inference is to avoid as far as possible, in our treatment of those afflicted with *Tænia solium*, such drugs as are likely to excite emesis. This is only important in the case of *Tœnia solium*, as the cysticercus of no other variety is known to infest the tissues of man.

The *Tœnia echinococcus* is a parasite of the dog, existing only in its larval state in the human system. It possesses great interest to the pathologist, chiefly on account of the fact that in one of its forms. *Echinococcuo multilocularis*, it was constantly mistaken for colloid cancer up to so late a period as 1856, when Virchow pointed out its true nature.

The so-called hydatid cysts are rarely met with in this country. But two specimens have been presented to the Pathological Society of Philadelphia, one by the late Dr. J. B. Mustin for Dr. Nancrede, recorded in vol. iii. of the Transactions; the other by Dr. Hutchinson, recorded in vol. iv.

An exceedingly rare specimen of *Tœnia*, the *Tœnia nana*, was exhibited by Dr. E. A. Spooner, of this city, before the College of Physicians in 1872. As far as I am aware, it had been previously observed only by Bilharz in Egypt. Heller, who had seen an account of Spooner's specimen in a German periodical, was inclined, in the absence of a minute description of the head of the animal, to regard it as a specimen of *Tœnia flavopunctata*, which it is said to resemble. In length, however, and in the number of its segments, Dr. Spooner's specimen accurately corresponds with the account given by Bilharz of the *Tœnia nana*. I am informed by Dr. Spooner that the patient who passed these worms has remained in good health ever since.

There are other intestinal parasites to which I would refer, did time permit. Among the most interesting of those remaining are the *Trichina spiralis* and the *Anchylostomum duodenale*. In countries where the latter prevails, the cachexia to which it gives rise is liable to be confounded with pernicious anæmia; and even in this country I would suggest that a careful search be made for this parasite in all autopsies in cases of supposed pernicious anæmia.

I have placed under the microscope the following specimens:—*Trichinæ*, free and in muscular tissue; *proglottis of Tœnia solium*, *Cysticercus cellulosæ*, *Acarus scabiei*, and *Echinococcus* brood capsules, for the use of which, as well as for that of the microscopes, I am indebted to Mr. Walmsley, of the firm of R. & J. Beck.—*Medical Times*.

